# Well-to-well transfer of HaloTag ligand 6-chlorohexan-1-ol and formaldehyde in a multi-well plate

**DOI:** 10.1038/s41598-025-29372-w

**Published:** 2025-11-29

**Authors:** Yugo Mishima, Saori Yasuda, Aoi Kunitomi, Tokiha Masuda-Ozawa, Shusuke Tomoshige, Shunji Kato, Yuki Kadono, Kiyotaka Nakagawa, Minoru Ishikawa

**Affiliations:** 1 Graduate School of Life Sciences, Tohoku University, 2-1-1 Katahira, Aoba-ku, Sendai, Miyagi 980-8577 Japan; 2 Graduate School of Agricultural Science, Tohoku University, 468-1 Aramaki Aza Aoba, Aoba-ku, Sendai, Miyagi 980-8572 Japan

**Keywords:** Multi-well plate, Volatilization, HaloTag, 6-chlorohexan-1-ol, Formaldehyde, Biochemistry, Chemical biology, Chemistry

## Abstract

When volatile organic compounds are handled in multi-well plates, their volatilization is known to affect non-treated wells in the plate. In this paper, we report two cases in which the HaloTag ligand 6-chlorohexan-1-ol and formaldehyde were volatilized and transferred to other wells in a multi-well plate. We found that competition between the HaloTag-targeting PROTAC and 6-chlorohexan-1-ol affected the other wells of the plate. A multifaceted analysis revealed that this phenomenon resulted from well-to-well transfer of 6-chlorohexan-1-ol into untreated wells on the plate. In addition, the formaldehyde used for cell fixation was toxic to cells in the surrounding wells. Therefore, even these reagents, which are widely used in life science research, require careful handling in multi-well plates.

**Supplementary Information:**

The online version contains supplementary material available at 10.1038/s41598-025-29372-w.

## Introduction

**Fig. 1:**
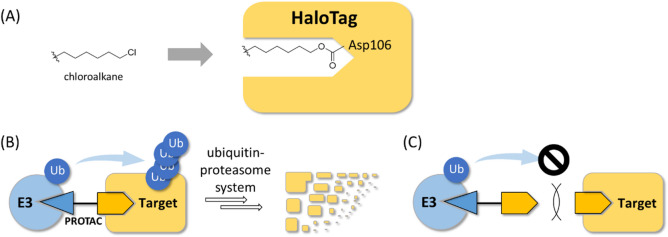
(**A**) Binding of chloroalkane to HaloTag; the carboxylic acid in HaloTag’s Asp106 reacts with the chloroalkane to form a covalent bond. (**B**) The mechanism by which PROTAC degrades target proteins. PROTAC enables the proximity of an E3 and the target protein, thereby facilitating the addition of ubiquitin (Ub) to the target by the E3 complex. The proteasome subsequently recognizes the ubiquitin chain, leading to the degradation of the target. (**C**) Competing for the PROTAC with an excess amount of the target ligand prevents the target from binding to the PROTAC and degradation.

HaloTag is a tag protein that can form a covalent bond with chloroalkane^1^ (Fig. 1A), and is often used in life science research. Recently, HaloTag has been used in proof-of-concept studies for targeted protein degraders, such as proteolysis-targeting chimeras (PROTACs)^2–5^. PROTACs have a structure that links the ligand of ubiquitin ligase (E3) to the ligand of the target protein, bringing the two proteins into proximity and inducing ubiquitination and proteasomal degradation of the target protein^6^ (Fig. 1B). Confirming that the degradation activity of PROTAC is canceled by competing with an excess amount of each protein ligand is important in analyzing the mode of action of the newly synthesized PROTAC. This competition assay is generally accepted in PROTAC research (Fig. 1C).

Multi-well plates are frequently used in life science research involving proteins and cells, including PROTAC development. Each well is generally recognized as an independent system. However, some compounds have been reported to affect other wells of the plate through volatilization. For instance, Adolphe et al. found that ethanol and butanol volatilize and are transferred to other wells^7^. Novy et al. reported that plant-derived compounds, such as thymoquinone, can diffuse in a plate and cause false positives in assays and proposed the use of an ethylene vinyl acetate (EVA) capmat as a vapor barrier to avoid this problem^8,9^. Castell et al. also observed the cross-contamination of phenol derivatives in a multi-well plate and reported that the use of plastic seals could prevent contamination without adversely affecting cell integrity^10^. These results indicate that assays using multi-well plates require attention to the volatilization of test compounds. However, this phenomenon has not received much attention because the reported compounds are not universally used in biological research.

In this study, we investigated the volatilization and transfer of 6-chlorohexan-1-ol between wells on multi-well plates. This compound is often used in life science research as a ligand for HaloTag, and its volatility is often underestimated owing to its high boiling point of 212.8 °C^11^. In addition, we tested the possibility that formaldehyde, which is used to fix cells and tissues^12^, could also affect the surrounding wells in a plate. If these commonly used compounds are transferred to other wells, researchers may encounter unexpected anomalies during the experiments. Therefore, we have shared this information in this study.

## Results

### HaloTag ligand 6-chlorohexan-1-ol-treated wells affect other wells in a multi-well plate

**Fig. 2:**
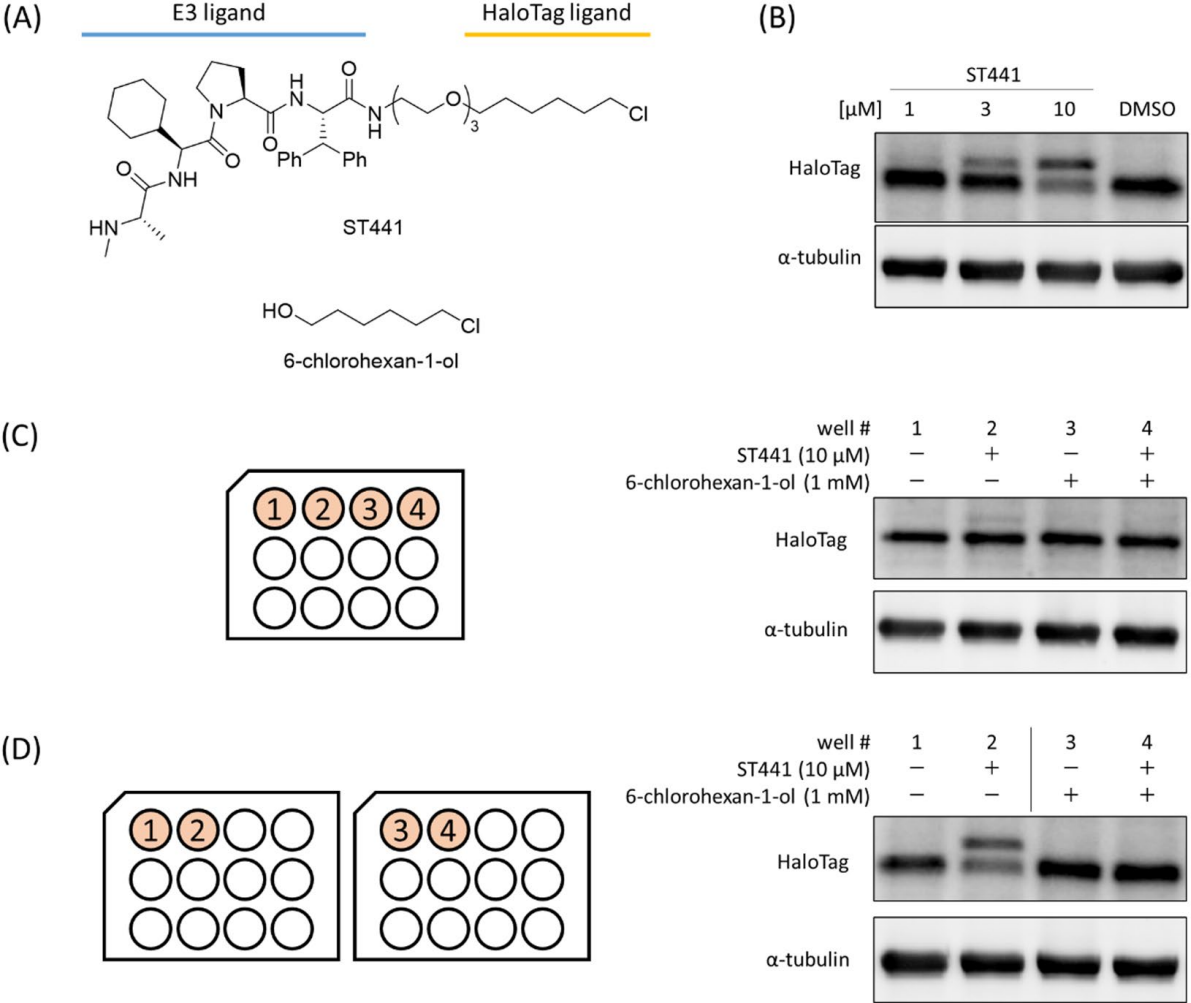
(**A**) The chemical structure of ST441 and 6-chlorohexan-1-ol. (**B**) A representative result of the evaluation of HaloTag-degradation activity of ST441. HEK293 cells expressing HaloTag were treated with ST441 at the indicated concentration for 24 h. The HaloTag and α-tubulin (a loading control) were detected by western blotting. A band shift and decrease in HaloTag level due to the covalent binding of ST441 and induced degradation was observed. (**C**) Competition experiments between ST441 and 6-chlorohexan-1-ol were performed in the same manner as in (**B**), using a single 12-well plate. (**D**) The same experiment as in (**C**) was performed using two 12-well plates. The blot images in panels (**B**–**D**) were created by cropping parts of the original images, and the original blots are presented in Supplementary Fig. 1.

We have previously reported that PROTAC ST441 degrades HaloTag or HaloTag-fused proteins^3^ (Fig. 2A, B). We selected 6-chlorohexan-1-ol (Fig. 2A) as the ligand to perform a competition assay between ST441 and a HaloTag ligand because it has been used as a HaloTag ligand in previous studies, including one related to HaloTag degraders^13^. However, when ST441 was allowed to compete with the HaloTag ligand 6-chlorohexan-1-ol, no degradation was observed under any conditions, including the well ST441-single treatment to HEK293 cells expressing HaloTag. We initially suspected technical problems with western blotting or cell conditions, but our attempts to improve them did not resolve this problem. Therefore, we suspected that 6-chlorohexan-1-ol was transferred to other wells, and HaloTag was blocked even in 6-chlorohexan-1-ol-untreated cells. First, we performed ST441 and 6-chlorohexan-1-ol competition assays in cells on a single plate or two separate plates to isolate the 6-chlorohexan-1-ol-treated wells and confirm that the presence of 6-chlorohexan-1-ol-treated wells in the plate affected the activity of ST441 in other wells. As expected, the results showed that when the experiment was performed on a single plate, the degradation of HaloTag was canceled, even in the ST441-treated well (Fig. 2C, well #2). In contrast, when the experiment was performed using separate plates, the degradation activity of ST441 was maintained (Fig. 2D, well #2).

### 6-chlorohexan-1-ol binds to HaloTag in an adjacent well

**Fig. 3:**
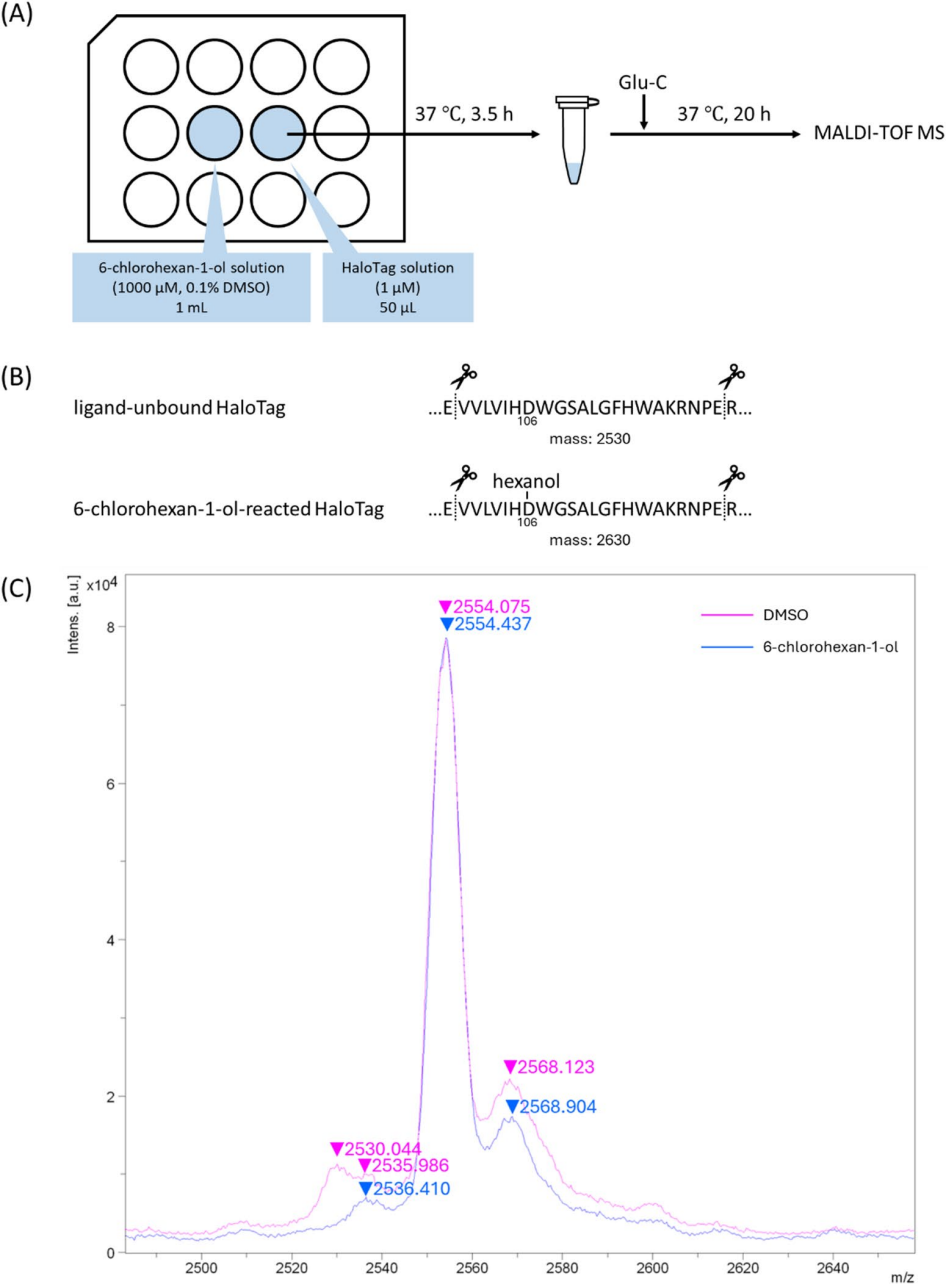
(**A**) The experimental procedure. (**B**) Sequence and mass of the fragment containing the ligand-binding residue (Asp106) from the Glu-C digestion products of ligand-unbound GST-HaloTag or 6-chlorohexan-1-ol-reacted GST-HaloTag. (**C**) MALDI-TOF MS results of digested fragments of GST-HaloTag from the adjacent wells treated with vehicle DMSO (magenta) or 6-chlorohexan-1-ol (blue).

Next, we detected the 6-chlorohexan-1-ol-bound HaloTag in wells not treated with 6-chlorohexan-1-ol using matrix-assisted laser desorption/ionization time-of-flight mass spectrometry (MALDI-TOF MS). A solution of 6-chlorohexan-1-ol (or vehicle DMSO) and GST-fused HaloTag (GST-HaloTag) solution were placed in adjacent wells of a 12-well plate and incubated at 37 °C for 3.5 h. The GST-HaloTag was then digested with Glu-C, a serine protease that cleaves the C-terminus of glutamic acid residues, and the digested fragments were analyzed using MALDI-TOF MS (Fig. 3A). As HaloTag covalently binds to its ligand via Asp106, it is expected to detect *m/z* 2530 of the intact V100-E121 fragment or *m/z* 2630 of its hexanol-adduct (Fig. 3B). Peaks *m/z* 2530 (HaloTag V100-E121), 2536 (unidentified), 2554 (from the GST tag), and 2568 (unidentified) were detected in GST-HaloTag fragments from plates containing the vehicle. However, the analysis of fragments obtained from plates containing 6-chlorohexan-1-ol showed no change in the *m/z* 2536, 2554, and 2568 peaks, whereas the *m/z* 2530 peak disappeared. (Fig. 3C). The disappearance of the peak at *m/z* 2530 suggested that 6-chlorohexan-1-ol was transferred and bound to the GST-HaloTag in the adjacent well. However, hexanol-bound fragments were not detected. This fragment may be less likely to be ionized, as similar results were obtained even in the positive control, in which the GST-HaloTag and 6-chlorohexan-1-ol were directly mixed (Supplementary Fig. 2).

**Fig. 4:**
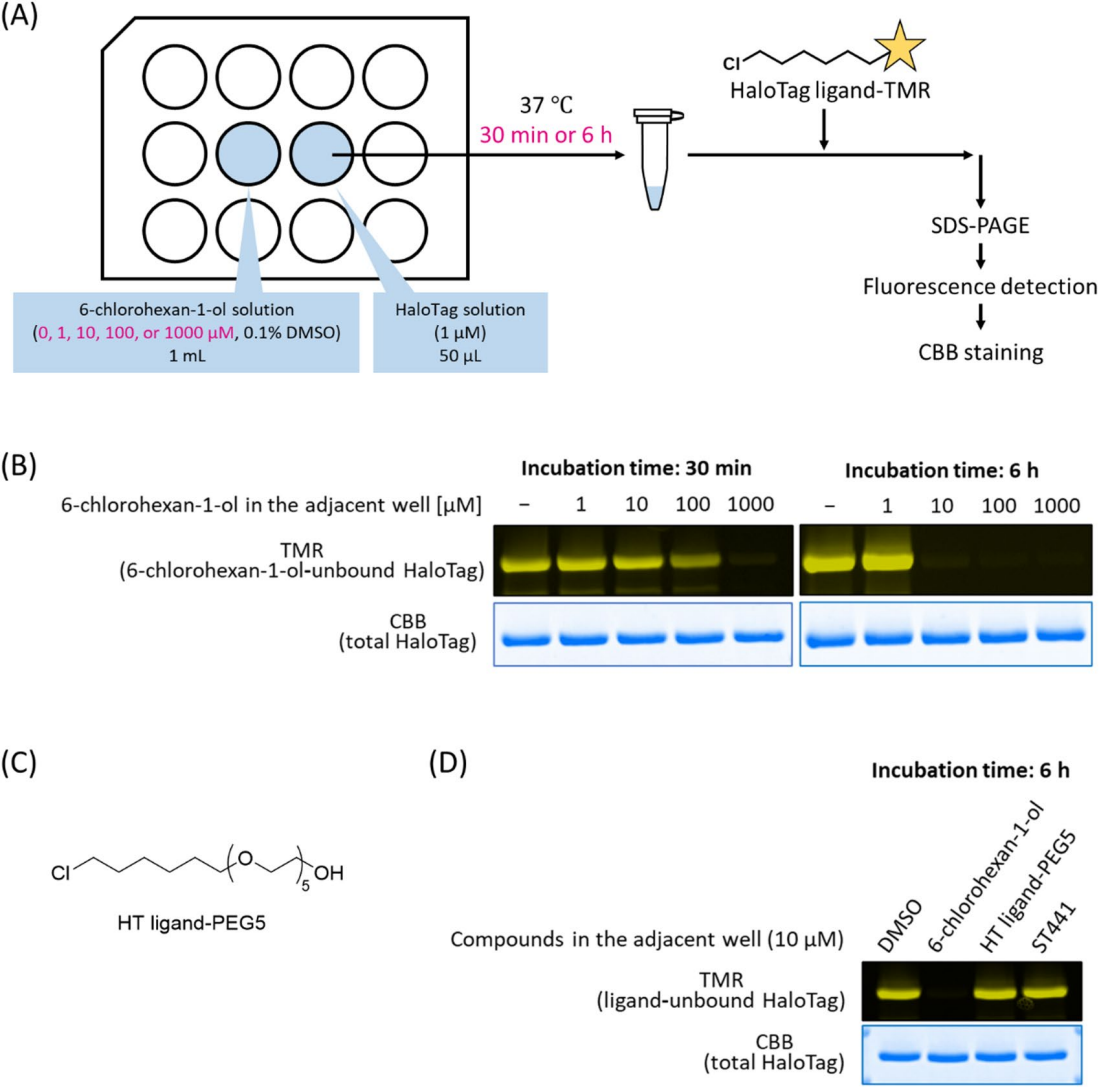
(**A**) The experimental procedure. (**B**) The results of SDS-PAGE. 6-Chlorohexan-1-ol-unbound GST-HaloTag was detected using TMR fluorescence, and total HaloTag was visualized using CBB staining. (**C**) The chemical structure of HT ligand-PEG5. (**D**) Results of the same experiments as (**A**) using 10 µM 6-chlorohexan-1-ol, HT-ligand-PEG5, or ST441. The gel images in panels (**B**) and (**D**) were created by cropping parts of the original images, and the original gels are presented in Supplementary Fig. 3.

To further confirm the 6-chlorohexan-1-ol transfer, 0, 1, 10, 100, or 1000 µM 6-chlorohexan-1-ol solution and recombinant GST-HaloTag solution were placed in the adjacent wells of a 12-well plate and incubated at 37 °C for 30 min–6 h. The resulting GST-HaloTag solution was incubated with the fluorescent HaloTag tetramethylrhodamine (TMR) ligand to label the 6-chlorohexan-1-ol-unbound GST-HaloTag, and the protein solution was subjected to sodium dodecyl sulfate-polyacrylamide gel electrophoresis (SDS-PAGE) (Fig. 4A). The results showed a concentration- and time-dependent decrease in fluorescently labeled GST-HaloTag, suggesting that 6-chlorohexan-1-ol bound to GST-HaloTag in adjacent wells in a concentration- and time-dependent manner (Fig. 4B). The tendency of 6-chlorohexan-1-ol to volatize and transfer to adjacent wells is thought to be due to its small molecular weight (MW: 137). On the other hand, relatively larger compounds such as a polyethylene glycol linked HaloTag ligand (HT ligand-PEG5, MW: 357, Fig. 4C) and ST441 (MW: 812) were assumed to have relatively low vapor pressure, so we used the same method to check whether these compounds would affect the adjacent wells. When 10 µM solutions of each compound were placed in adjacent wells for 6 h, 6-chlorohexan-1-ol bound to HaloTag in the adjacent well, but HT ligand-PEG5 and ST441 did not appear to transfer (Fig. 4D). These results suggest that this transfer would not occur if 6-chlorohexan-ol were linked to some larger structure, and a polyethylene glycol-linked compound rather than 6-chlorohexan-1-ol would be preferable as a competitor for the HaloTag-targeting PROTACs.

### Quantification of the 6-chlorohexan-1-ol transfer

**Fig. 5:**
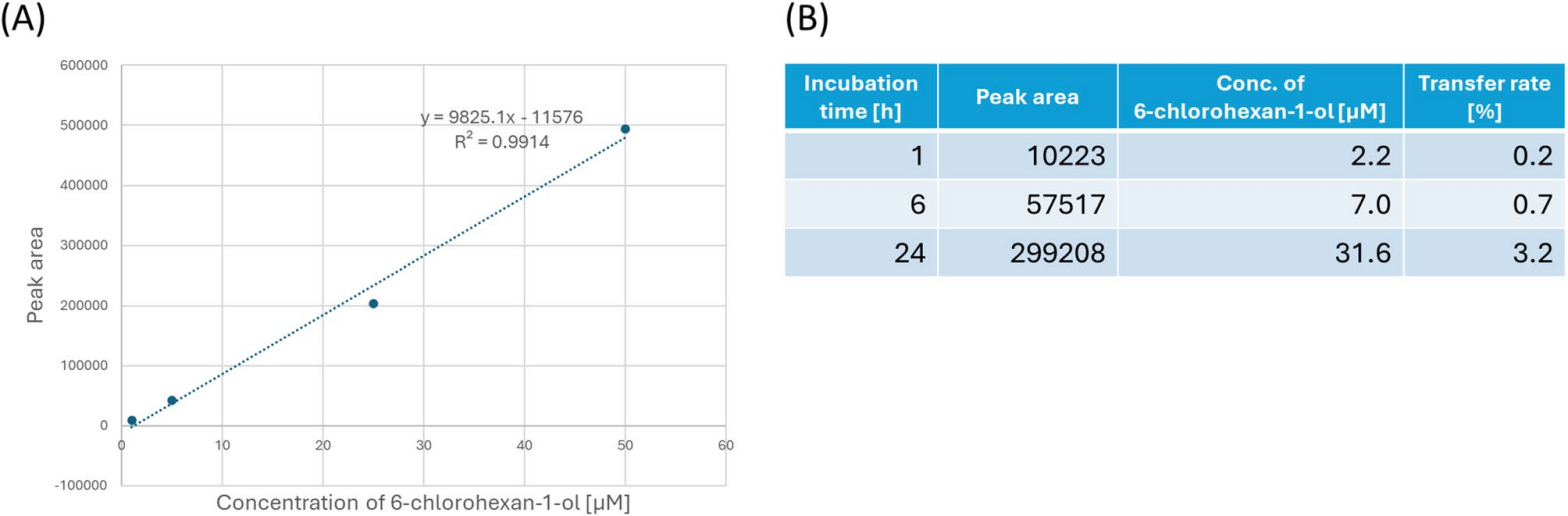
(**A**) The calibration curve prepared from 1, 5, 25, and 50 µM 6-chlorohexan-1-ol standards. (**B**) Calculated concentration and transfer rate of 6-chlorohexan-1-ol from the calibration curve.

Quantification was performed using gas chromatography-mass spectrometry (GC-MS) to determine the transfer rate of 6-chlorohexan-1-ol to an adjacent well. First, the vapor of the 6-chlorohexan-1-ol solution was analyzed using GC-MS, and the compound was identified using spectral comparison with a mass spectral library to determine the retention time of 6-chlorohexan-1-ol (Supplementary Fig. 4). Aqueous 6-chlorohexan-1-ol solutions of known concentrations were analyzed, and a calibration curve was constructed from their peak areas (Fig. 5A). Subsequently, the concentration of 6-chlorohexan-1-ol in pure water that had been incubated for 1, 6, or 24 h in the well adjacent to the 1 mM 6-chlorohexan-1-ol solution was calculated from the calibration curve. The results showed that the transfer rate increased in a time-dependent manner, with 0.2% 6-chlorohexan-1-ol transferred to the adjacent well at 1 h, 0.7% at 6 h, and 3.2% at 24 h (Fig. 5B).

### Formaldehyde-treated wells affect other wells in a multi-well plate

**Fig. 6:**
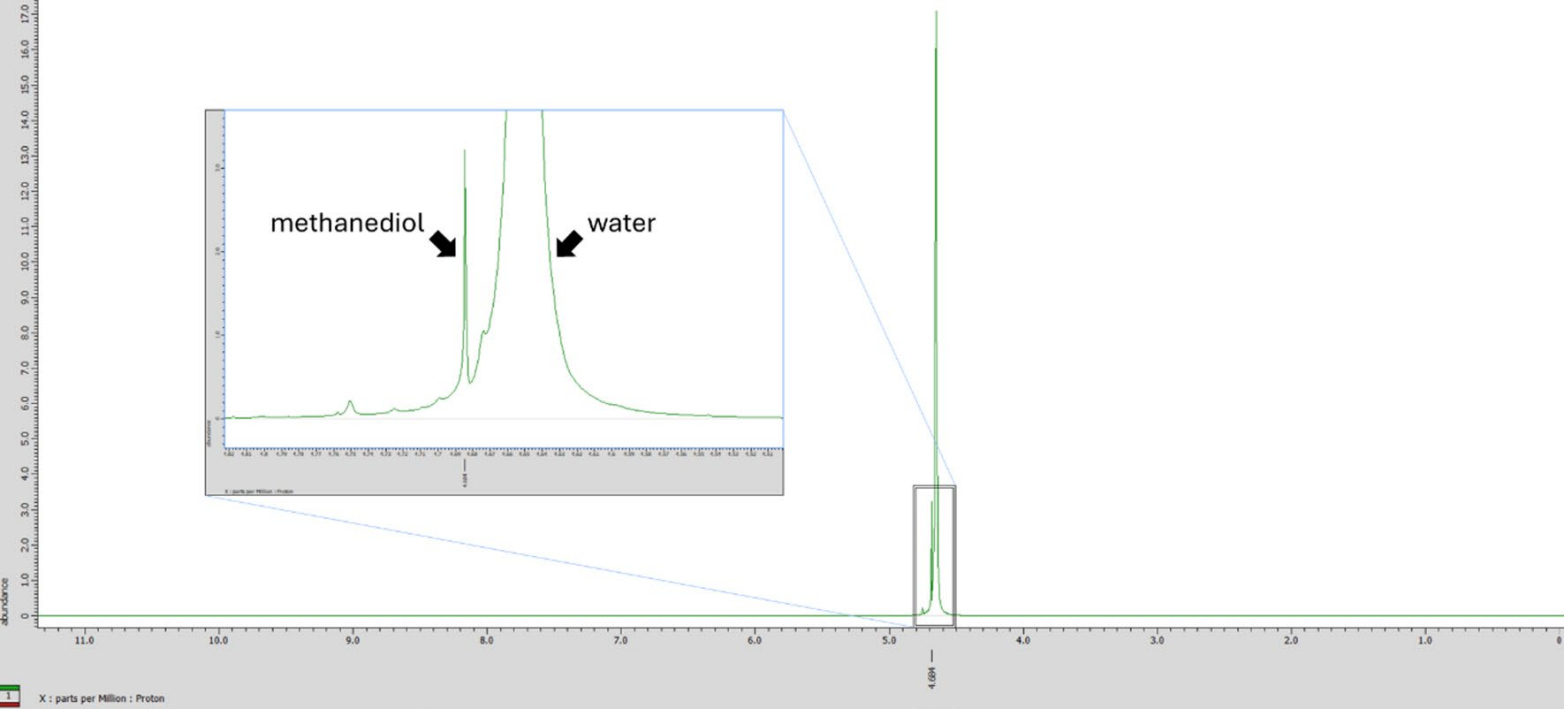
^1^H-NMR chart of D_2_O placed in the adjacent well of 4% PFA solution.

During one experiment, we fixed some cells using 4% paraformaldehyde solution (4% PFA) in a multi-well plate for microscopic observation but later noticed that the unfixed surrounding cells had died. We suspected that formaldehyde had volatilized and transferred into the neighboring wells of the plate. To confirm formaldehyde transfer, 4% PFA and deuterium oxide (D_2_O) were placed in adjacent wells of a 12-well plate, and the D_2_O was analyzed using proton nuclear magnetic resonance (^1^H-NMR) after 24 h of incubation. The NMR spectrum showed a peak corresponding to methanediol, a product of the hydration of formaldehyde (Fig. 6). This result indicated that formaldehyde (and possibly methanediol) was transferred between the wells.

**Fig. 7:**
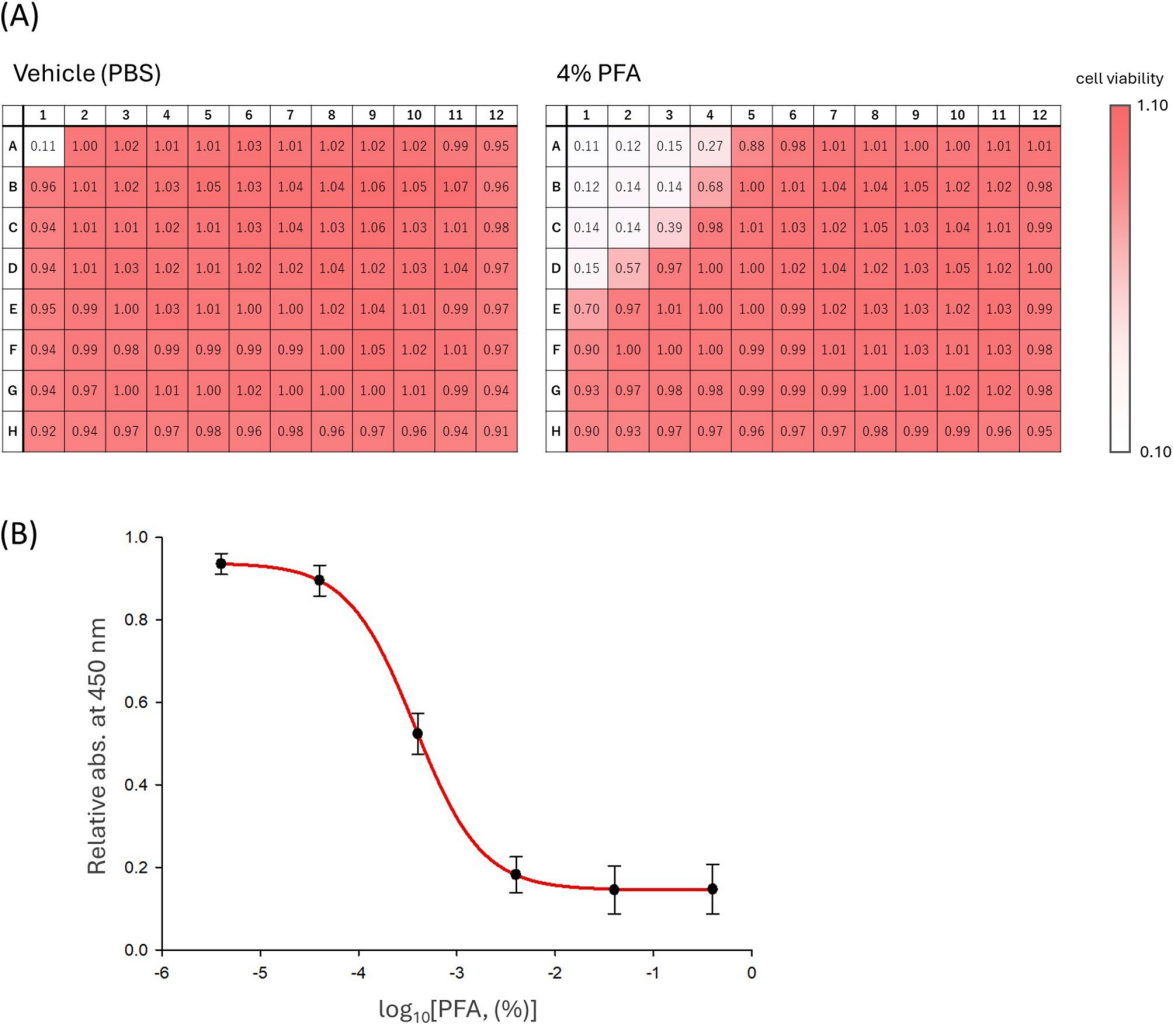
(**A**) HeLa cells were seeded on a 96-well plate, and the A1 well was treated with vehicle (PBS) or 4% PFA for 20 min. The solution was then removed, and the well was washed. Cell viability was evaluated using a Cell Counting Kit-8 after 24 h of culture. Values are presented relative to the mean absorbance at 450 nm of WST-8 formazan in all wells except A1 of the vehicle-treated plate, which was set to 1. Data are the mean from three independent experiments. (**B**) HeLa cells were treated with serial dilutions of 4% PFA ranging from 10-fold to 10^6^-fold. After 24 h of incubation, cell viability was evaluated using a Cell Counting Kit-8. Data are shown as mean ± SE relative to the mean absorbance of formazan in the control wells (treated with PBS), which was set to 1.

Next, we examined the extent to which formaldehyde diffusion during the 20-min incubation required for cell fixation affected the wells within the plate. The A1 well of a 96-well plate containing seeded HeLa cells was treated with 4% PFA (or vehicle PBS) for 20 min. The A1 well was then washed with PBS and emptied, and cell viability in the plate was measured using the Cell Counting Kit-8 after 24 h of culture. As a result, in plates treated with the vehicle, all wells except the emptied A1 well supported normal cell growth, whereas in plates treated with 4% PFA, cell proliferation was suppressed in the vicinity of the A1 well. From these results, it was confirmed that a 20-min incubation induced cytotoxicity within a circular area corresponding to a radius of approximately five wells (Fig. 7A). We further aimed to quantify the amount of formaldehyde that transferred into the adjacent well during the 20-min incubation with 4% PFA. To this end, 4% PFA was added to the A1 well of a 96-well plate and D_2_O to the A2 well, followed by a 20-min incubation. After incubation, a defined amount of DMSO was added to the D_2_O in the A2 well as an internal standard, and the concentration was calculated from the ratio of the DMSO signal to the methanediol signal in the ^1^H-NMR spectrum. However, no methanediol signal was detected after the 20-min incubation (Supplementary Fig. 5). Considering that formaldehyde may exert cytotoxic effects at concentrations too low to be detected by NMR, we treated cells with serial dilutions of 4% PFA ranging from 10-fold to 10^6^-fold and measured cell viability after 24 h. As a result, compared with the control (PBS), cell viability was reduced by approximately half at 4 × 10^−4^% PFA, and nearly all cells were killed at concentrations of 4 × 10^−3^% or higher (Fig. 7B). From these findings, we infer that in the experiment shown in Fig. 7A, the solution in the adjacent wells contained an amount of formaldehyde exceeding that corresponding to 4 × 10^−4^% PFA. Although the amount of formaldehyde transferred during the 20-min incubation was minimal, it was likely sufficient to cause cytotoxicity.

## Discussion

**Fig. 8:**
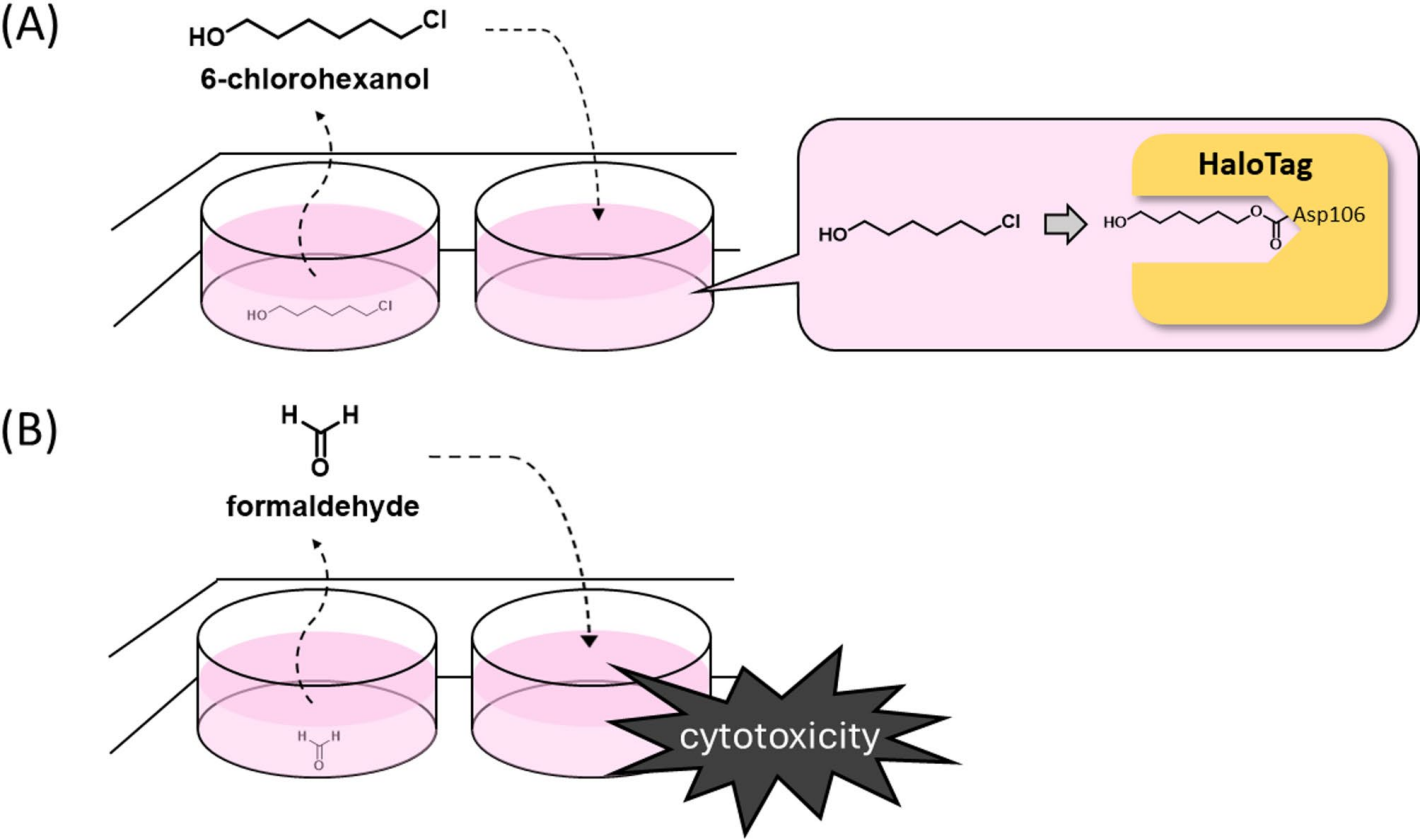
Well-to-well transfer of 6-chlorohexan-1-ol (**A**) and formaldehyde (**B**) in a multi-well plate.

This study showed that the HaloTag ligand, 6-chlorohexan-1-ol, was volatilized in a multi-well plate and transferred to other wells in a concentration- and time-dependent manner (Fig. 8A). This compound has been used as a HaloTag ligand; therefore, this phenomenon may have skewed the assay results and confused some researchers. Therefore, when experiments are conducted in multi-well plates, wells treated with 6-chlorohexan-1-ol should be separated or vapor barriers should be used. Alternatively, a nonvolatile compound such as HT ligand-PEG5 should be selected instead of 6-chlorohexan-1-ol as the HaloTag ligand. Indeed, we previously performed ligand competition experiments without problems by using a polyethylene glycol-linked chloroalkane^3^. Formaldehyde is often used to fix cells and tissues; however, it also volatilizes in multi-well plates and affects the viability of cells in other wells (Fig. 8B). If one wishes to fix only certain cells during an experiment, it is necessary to seed the cells on different plates beforehand. During the course of our study, a similar study was independently published by another group, which reported that formaldehyde affects signaling in cells in neighboring wells^14^. This report and our results are mutually supportive, and we further discussed the spatial extent to which formaldehyde affects cell viability.

Volatile solutes partition between the gas and liquid phases according to Henry’s law. Within a plate, solutes that volatilize diffuse through the gap between the plate and its lid, and subsequently re-dissolve in neighboring wells. Referring to the Henry’s law constant of a compound is considered useful for predicting its potential to diffuse within a plate. The Henry’s law constant of formaldehyde in aqueous solution is 3.41 × 10^−2^ Pa·m^3^/mol at 25 °C^15^, and this value can serve as a reference for estimating the likelihood of well-to-well transfer. However, the Henry’s law constants of all compounds are not known, and no experimental value for the Henry’s law constant of 6-chlorohexan-1-ol could be found. To estimate the possibility of well-to-well transfer based on easily accessible or measurable physical properties, analyses of a larger set of compounds are required. Nevertheless, since the Henry’s law constant is known to approximate the ratio of vapor pressure to aqueous solubility, particularly for compounds with low solubility^16^, it can be said that compounds with high vapor pressure and low water solubility warrant special attention regarding their potential for well-to-well transfer.

## Methods

### Cell culture

HEK293 cells stably expressing HaloTag (established in our previous research^3^) and HeLa cells (RCB0007; RIKEN BRC, the National BioResource Project of the MEXT/AMED, Japan) were cultured in Dulbecco’s modified Eagle’s medium (DMEM) supplemented with 5% (for HEK293) or 10% (for HeLa) heat-inactivated fetal bovine serum (FBS) and penicillin/streptomycin at 37 °C in a humidified atmosphere containing 5% CO_2_ in air.

### Western blotting

5 × Laemmli buffer (0.25% bromophenol blue (BPB), 0.5 M dithiothreitol (DTT), 50% glycerol, 10% sodium dodecyl sulfate (SDS), 0.25 M Tris–HCl (pH 6.8)) was added to the cell lysate and the mixture was heated at 95 °C for 5 min and then cooled on ice. Proteins were separated using SDS-PAGE with SuperSep Ace 10–20%, 17-well gels (198-15041; FUJIFILM Wako Pure Chemical Corporation, Osaka, Japan) and transferred onto Immun-Blot polyvinylidene difluoride (PVDF) membrane (1620264; Bio-Rad Laboratories, Hercules, CA, USA). After blocking with Bullet Blocking One for Western Blotting (13779-01; Nacalai Tesque, Kyoto, Japan) for 15 min, the membrane was probed with an antibody, as shown in Table 1. Antibodies were diluted with Can Get Signal Immunoreaction Enhancer Solution 1 or 2 (NKB-101; TOYOBO, Osaka, Japan). The membrane was rinsed with water (five times) and washed with TBS-T for 5 min, and the immunoblots were visualized by measuring the enhanced chemiluminescence with ImmunoStar LD (292-69903; FUJIFILM Wako Pure Chemical Corporation) or the fluorescence from fluorescent-dye conjugated antibodies using a ChemiDoc MP Imaging System (Bio-Rad Laboratories). Images were analyzed using Image Lab software (Bio-Rad Laboratories).

**Table 1:** Antibodies used for Western blotting.

Primary antibody			Secondary antibody		
Antigen	Dilution ratio	Cat. No.	Antigen	Dilution ratio	Cat. No.
HaloTag	1000:1	G928A^*1^	Rabbit IgG	5000:1	5220 –0458^*2^
α-tubulin	2000:1	12,004,166^*3^	-	–	–

^*1^Promega, WI, USA ^*2^ SeraCare, MA, USA ^*3^ Bio-Rad laboratories.

### Evaluation of Degradation Activity of ST441

HEK293 cells stably expressing HaloTag were seeded at a concentration of 2.0 × 10^5^ cells/mL in 12-well plates and incubated for 24 h. Then, the cells were cultured in medium containing ST441 at the indicated concentrations with 0.1% DMSO for 24 h. The cells were lysed on ice for 30 min with TNE-T lysis buffer (50 mM Tris-HCl (pH 7.4), 150 mM NaCl, 1% Triton X-100, and 5 mM ethylenediaminetetraacetic acid (EDTA)) supplemented with a 1% protease inhibitor cocktail (25955-24; Nacalai Tesque). The lysates were centrifuged at 16,710 × *g* and 4 °C for 5 min, and the supernatants were collected. The protein concentration in the supernatant was determined using the BCA protein assay and normalized. The amount of intracellular proteins was determined by western blotting.

### Competition between ST441 and 6-chlorohexan-1-ol

6-Chlorohexan-1-ol (1 mM) or vehicle was administered 1 h before ST441 treatment, and the same procedure as described above was performed using a single 12-well plate or two plates to isolate the 6-chlorohexan-1-ol-containing wells (0.2% DMSO).

### Plasmid construction for GST-HaloTag expression

For GST-HaloTag expression in bacteria, HaloTag DNA sequence was amplified from pFN21AB5414 (HaloTag-CREB1, Kazusa DNA Research Institute, Chiba, Japan) with primers containing restriction enzyme sites (BamHI-HaloTag-FW 5′-TTTGGATCCATGGCAGAAATCGGTACTGG-3′ [29mer, Tm = 60 °C, 48% GC] and HaloTag-EcoRI-RV 5′-TTTGAATTCTTAGCCGGAAATCTCGAGCGTC-3′ [31mer, Tm = 60 °C, 45% GC]) using KOD FX Neo polymerase (KFX-201; TOYOBO). HaloTag fragment and pGEX4T-1 were treated with BamHI and EcoRI-HF (R0136 and R3101; New England Biolabs, Ipswich, MA, USA). HaloTag coding sequence was cloned downstream of the thrombin site of the pGEX4T-1 plasmid by using T4 DNA ligase (2011 A; Takara Bio, Shiga, Japan). The sequence was confirmed by Sanger sequencing (Eurofins Genomics, Tokyo, Japan).

### Expression and purification of GST-HaloTag

GST-HaloTag protein was expressed in *Escherichia coli* BL21(DE3) cells (Nippon Gene, Tokyo, Japan). Cells were transformed with pGEX4T-1-HaloTag. Transformed cells were cultured in LB medium containing 0.1 mg/mL of ampicillin at 37 °C until the O.D. 600 reached 0.5–0.8. Expression was induced with 0.1 mM isopropyl-β-D-thiogalactopyranoside (IPTG) at 20 °C overnight. Collected cells were resuspended in PBS and lysed by sonication (SFX150HH; Emerson Electric Co., St. Louis, MO, USA). The lysate was clarified by centrifugation at 26,900 × *g* for 30 min at 6 °C and passed through a low protein binding PVDF 0.22 μm filter unit (SLGVR33RS; Merck Millipore Ltd., Ireland), then applied to GSTrap FF 5 mL (Cytiva, Tokyo, Japan) pre-equilibrated with PBS. The column was washed with 7 CV of PBS, then GST-HaloTag protein was eluted with 7 CV of GE buffer (10 mM reduced glutathione, 20 mM Tris-HCl in PBS). Glutathione was removed by dialysis. Purified protein was concentrated with Amicon-Ultra-0.5 (10 K MWCO) (Merck Millipore Ltd.) with PBS. Concentration of the protein was determined with the molar absorption coefficient^17^ ε280 = 102,050 M^−1^ cm^−1^. Absorbance at 280 nm was measured by using NanoDrop (Thermo Fisher Scientific, Waltham, MA, USA).

### Detection of ligand binding using MALDI-TOF MS

GST-HaloTag was dissolved in phosphate buffer (0.2 M sodium dihydrogen phosphate dihydrate, 0.2 M disodium hydrogen phosphate dodecahydrate) at a concentration of 1 µM. A 6-chlorohexan-1-ol or vehicle aqueous solution (1000 µM, 1 mL, 0.1% DMSO) and the GST-HaloTag solution were placed in the B2 and B3 wells, respectively, of a 12-well plate. Simultaneously, the GST-HaloTag solution and 6-chlorohexan-1-ol were mixed directly into a microtube as a positive control. The plates and the tube were incubated at 37 °C for 3.5 h. Each resulting HaloTag solution was transferred into a microtube and treated with Glu-C (stock: 0.25 µg/µL, 0.8 µL) for 20 h at 37 °C to digest the protein. The GST-HaloTag digested fragment solution was desalted using ZipTip with 0.6 µL C_18_ resin (Merck KGaA, Darmstadt, Germany) and dried at room temperature on a MALDI-TOF MS plate. The α-cyano-4-hydroxycinnamic acid (CHCA) solution was then placed on top of it and dried in the same manner, and the digested fragments were detected by MALDI-TOF MS analysis using autoflex speed (Bruker, Billerica, MA, USA).

### Binding inhibition of fluorescent halotag ligands

In a 12-well plate, 1 mL of aqueous solution of 6-chlorohexan-1-ol, HT ligand-PEG5, or ST441 (0.1% DMSO) was placed in the B2 well and 50 µL of binding buffer (25 mM HEPES (pH 7.5), 50 mM KCl, 0.01% TritonX-100) containing GST-HaloTag (1 µM) was placed in the B3 well. After incubation in a CO_2_ incubator at 37 °C for 30 min or 6 h, 20 µL of the solution from the B3 well was aliquoted into microtubes and 1 µL of 50 µM HaloTag TMR Ligand (G8252; Bio-Rad Laboratories) was added and incubated in the dark at room temperature for 1 h. SDS-PAGE was then performed as described in “Western Blotting” to detect the fluorescent ligand, followed by Coomassie Brilliant Blue (CBB) staining. Gels were photographed using a ChemiDoc MP Imaging System (Bio-Rad Laboratories).

### Quantification of 6-chlorohexan-1-ol transfer

In a 12-well plate, 1 mL of 6-chlorohexan-1-ol aqueous solution (0.1% DMSO) was placed in well B2, and 1 mL of water was placed in well B3. After incubation at 37 °C for the indicated times, the water in the B3 well was analyzed by GC-MS, as described below. The standard aqueous solutions of 6-chlorohexan-1-ol (1, 5, 25, and 50 µM) were also prepared and analyzed by GC-MS for preparation of the calibration curve. The amount of 6-chlorohexan-1-ol was quantified based on the peak areas of its fragment ion (*m/z* 55).

### GC-MS analysis

6-Chlorohexan-1-ol was extracted using solid-phase microextraction (SPME) (Supelco, Inc., Bellefonte, PA, USA) at 40 °C for 15 min. The SPME fiber was kept 1 cm above the sample to expose it to the headspace of the vial containing 700 µL of the solution. Later, the fiber was immediately desorbed into the GC-MS injection port at 250 °C for 4.5 min. Analysis was conducted using a GC-EI-MS system (GCMS-QP2010 SE; Shimadzu, Kyoto, Japan). The samples were separated by a DB-WAX-UI column (length: 60 m, internal diameter: 0.250 mm, film thickness: 0.25 μm; Agilent Technologies, Santa Clara, CA, USA). As a mobile phase, helium gas was used (25 cm/sec) with a gradient profile of 30 °C for 10 min, 30–250 °C at an increase of 5 °C/min, and 250 °C for 5 min. The scan range and ion source temperature were set at *m/z* 10–300 and 250 °C, respectively. Compounds were identified by spectral comparison with the NIST 17 mass spectral library using GCMS solution software (ver. 4.50).

### Verification of the extent of formaldehyde diffusion in a 96-well plate

HeLa cells were seeded at a concentration of 0.5 × 10^4^ cells/100 µL in 96-well plates and incubated for 1 h. Thereafter, the medium in the A1 well was exchanged with 4% PFA or vehicle PBS (100 µL) and incubated in the CO_2_ incubator (37 °C) for 20 min. The solution in the A1 well was removed and washed thrice with PBS (200 µL), then the A1 well was emptied. After 24 h of incubation, medium was removed from all wells and 100 µL of medium containing Cell Counting Kit-8 (CCK8) reagent (CK04; DOJINDO, Kumamoto, Japan) was added to each well (medium: CCK8, 10:1, v/v). After incubation in the CO_2_ incubator for 1.5 h, the absorbance was measured at 450 nm using an Envision 2104 multilabel reader (PerkinElmer, Inc., Waltham, MA, USA), and cell viability was evaluated.

### Evaluation of the cytotoxicity of PFA solutions at various concentrations

HeLa cells were seeded at a concentration of 0.5 × 10^4^ cells/100 µL into the D5, D6, and D7 wells of a 96-well plate. After 1 h, the cells were treated with serially diluted PFA solutions at final concentrations ranging from 4 × 10^−1^% to 4 × 10^−6^% (10% final PBS), and then cultured for 24 h in a CO_2_ incubator (37 °C). Cell viability was subsequently evaluated using the method described above.

### NMR analysis of transferred formaldehyde (12-well plate, 24 h incubation)

The PFA was diluted with D_2_O (to prevent H_2_O contamination due to transfer between wells) to a final concentration of 4%. In a 12-well plate, 750 µL of the 4% PFA D_2_O solution and pure D_2_O were placed in the B2 and B3 wells, respectively. After incubation at 37 °C for 24 h, the D_2_O in the B3 well was subjected to ^1^H-NMR, which was performed on a JEOL JNM ECA-600 spectrometer (JEOL, Tokyo, Japan).

### NMR analysis of transferred formaldehyde (96-well plate, 20 min incubation)

In six 96-well plates, 100 µL of 4% PFA was added to the A1 wells, 100 µL of D_2_O to the A2 wells, and 100 µL of H_2_O to the remaining wells, followed by incubation at 37 °C for 20 min. Subsequently, the D_2_O was completely collected from the A2 wells (a total of 600 µL), to which 1.18 mg of DMSO was added and mixed, and the sample was subjected to ^1^H-NMR analysis using JEOL JNM ECA-600 spectrometer (JEOL).

## Supplementary Information

Below is the link to the electronic supplementary material.

Supplementary Material 1

## Data Availability

The authors declare that the data supporting the findings of this study are available within the paper and its Supplementary Information files. Should any raw data files be needed in another format they are available from the corresponding author upon reasonable request.
