# Supplementary material for: Well-to-well transfer of HaloTag ligand 6-chlorohexan-1-ol and formaldehyde in a multi-well plate
Source: Sci Rep. 2025 Nov 29;16:67. doi: 10.1038/s41598-025-29372-w (PMC12764959; doi:10.1038/s41598-025-29372-w)
Supplement: Supplementary file 1 — Supplementary Material 1 [file 41598_2025_29372_MOESM1_ESM.pdf]

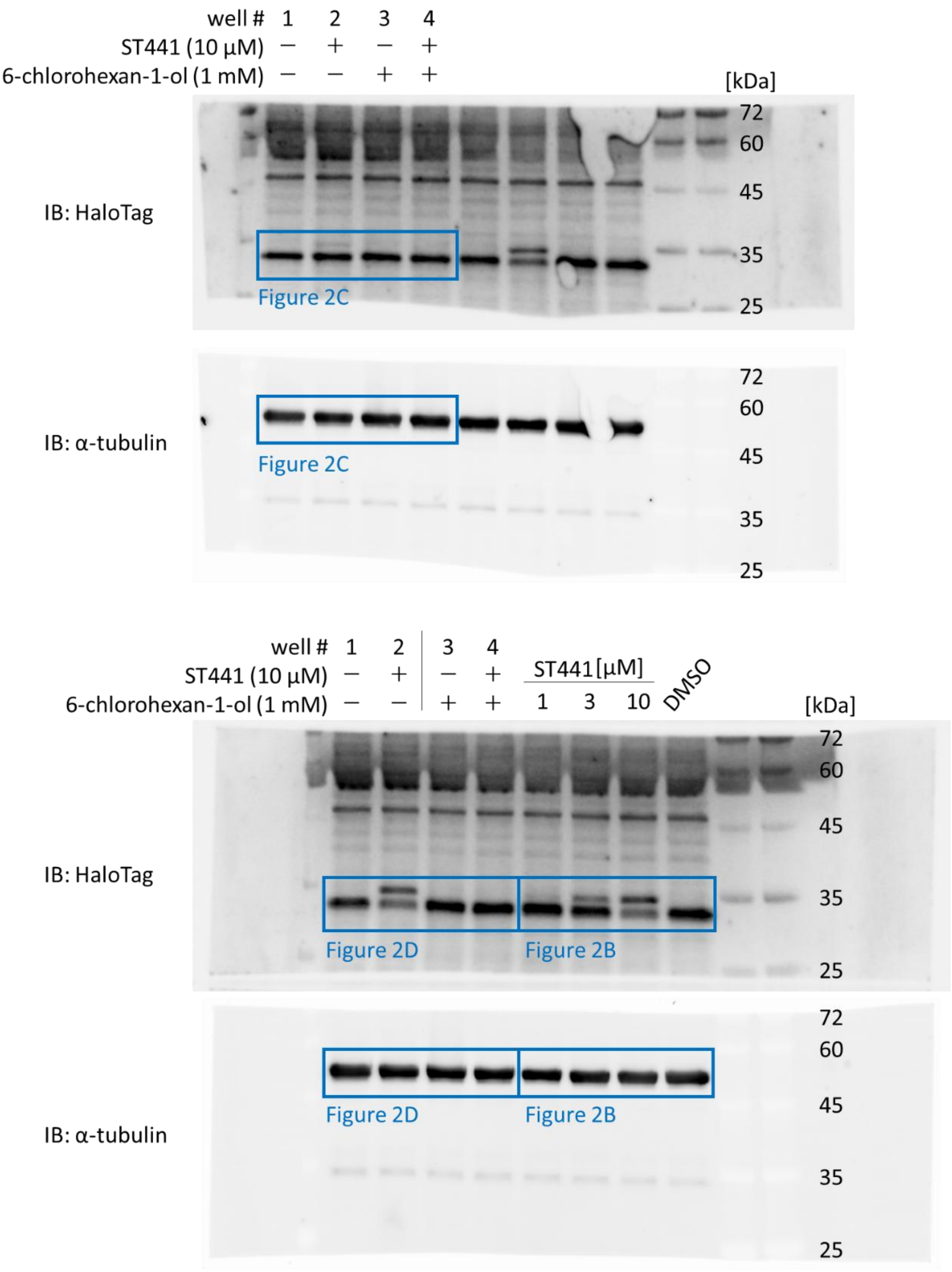

Supplementary Figure 1

Original blot images in Figure 2. The bands shown in Figure 2 are surrounded by blue squares.

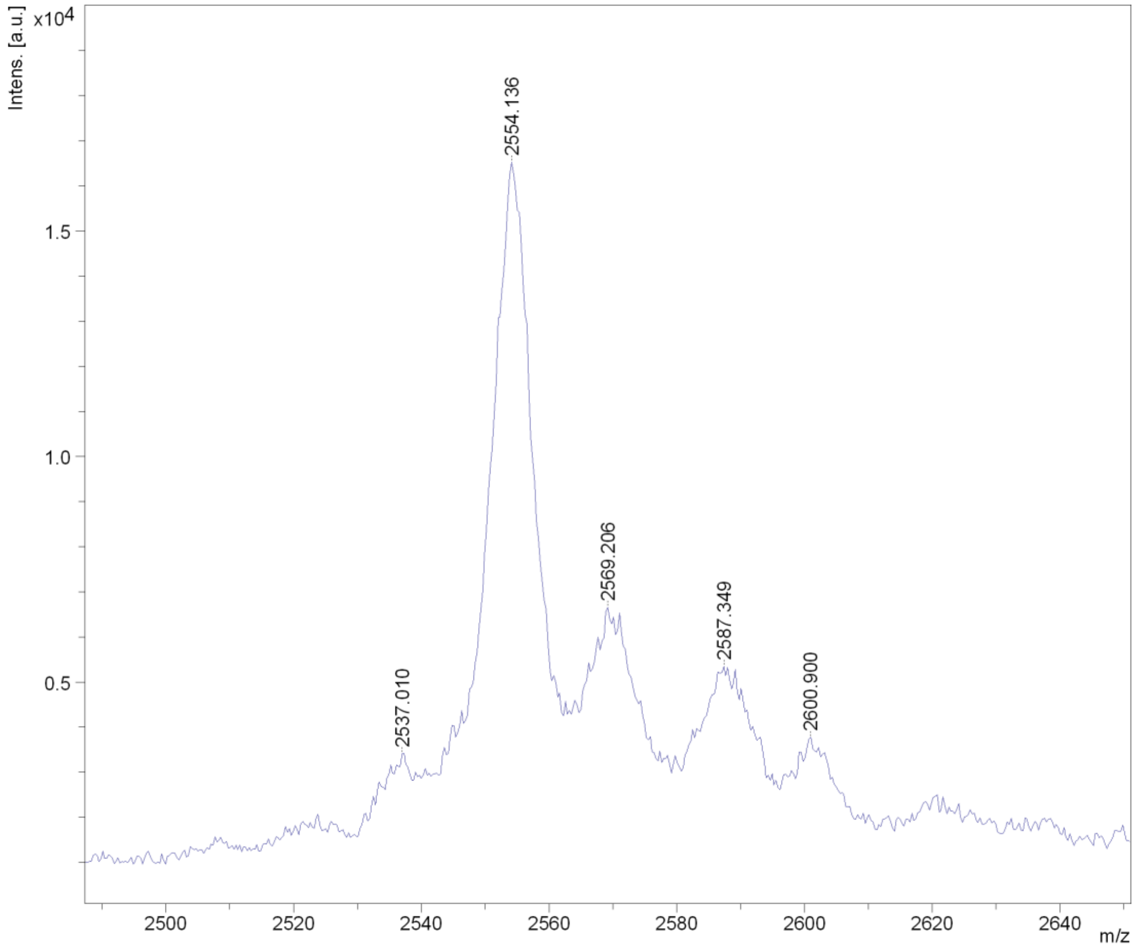

Supplementary Figure 2

MALDI-TOF MS analysis of the positive control in which HaloTag and 6-chlorohexan-1-ol were directly mixed. Although no hexanol-bound fragments were observed, the peak at *m/z* 2530 disappeared.

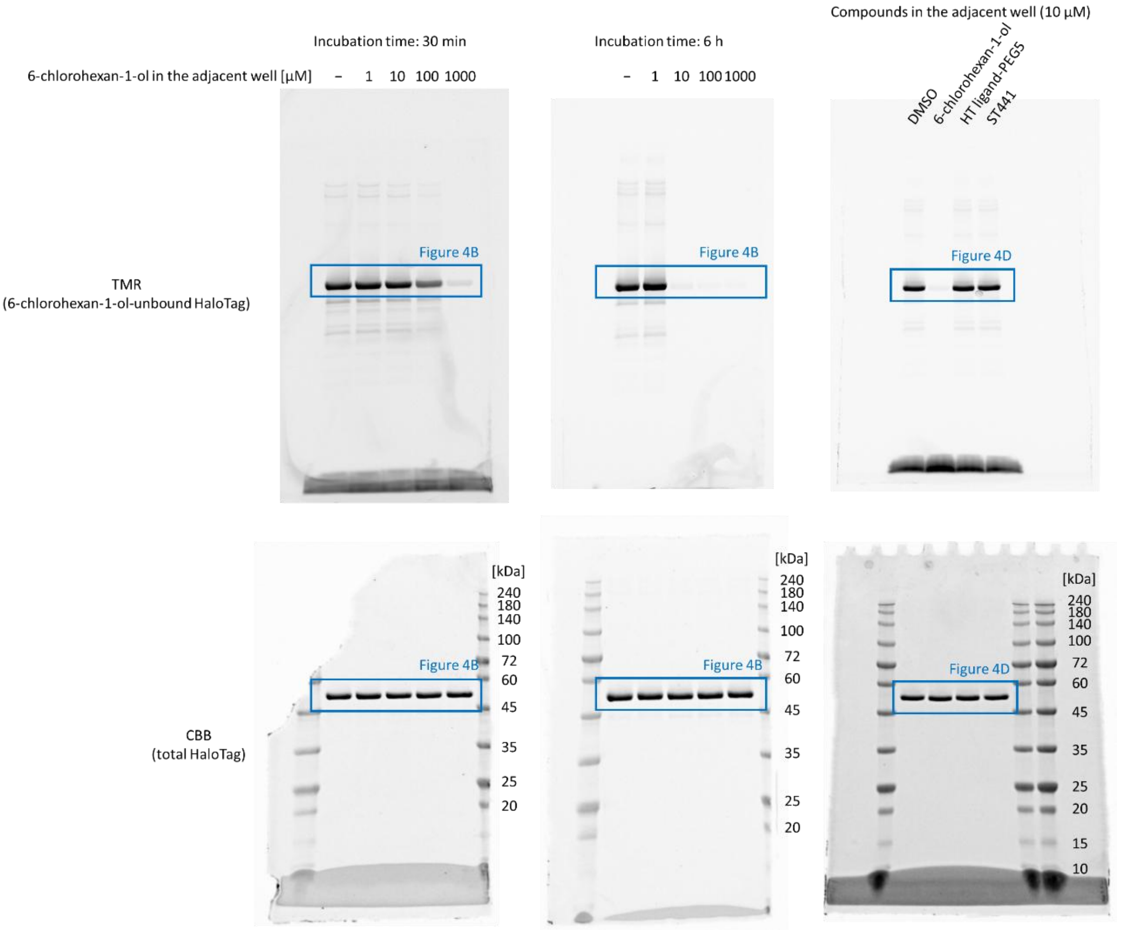

Supplementary Figure 3

Original gel images in Figure 4. The bands shown in Figure 4 are surrounded by blue squares.

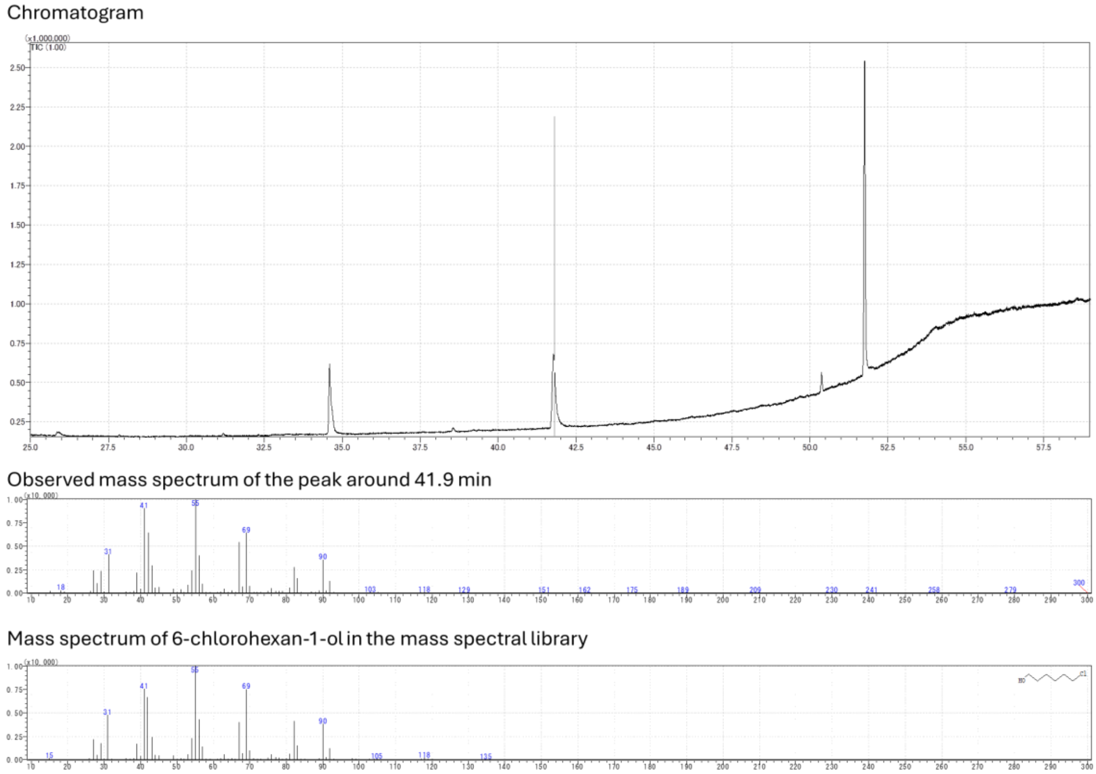

Supplementary Figure 4

Chromatogram and mass spectrum of a 6-chlorohexan-1-ol solution. The peak at a retention time of 41.9 min was identified as 6-chlorohexan-1-ol based on the mass spectrum library. The peak around 34.5 min was identified as 1,6-dichlorohexane, the peak around 50.4 min as 2,4-di-*tert*-butylphenol, and the peak around 51.8 min as diethyl phthalate.

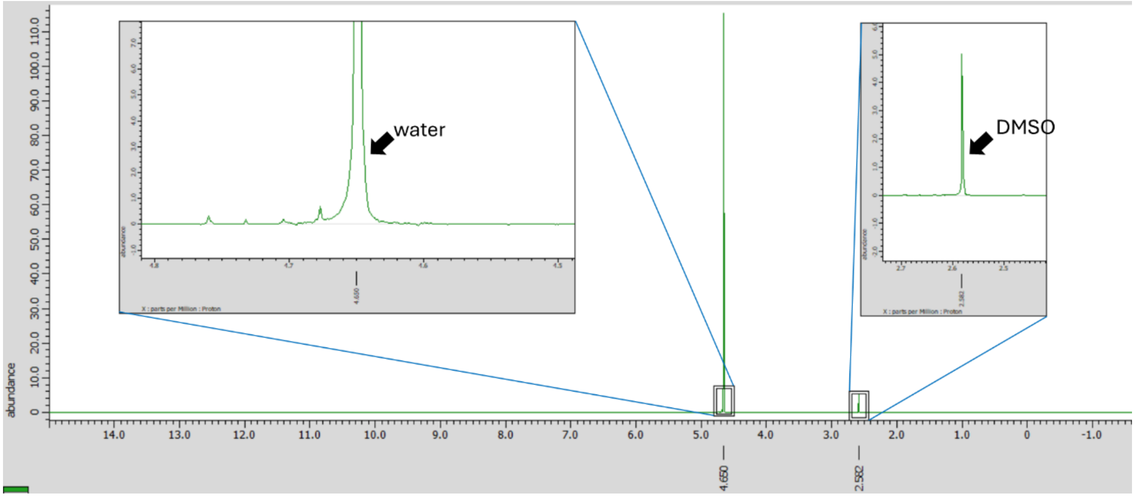

Supplementary Figure 5

^{1}H-NMR spectrum of D_2O placed in the well adjacent to 4% PFA for 20 min in a 96-well plate.
